# Loop Ileostomy With Colonic Lavage: Case Report of an Alternative to Total Colectomy in the Setting of Fulminant Clostridium difficile Colitis

**DOI:** 10.7759/cureus.73141

**Published:** 2024-11-06

**Authors:** Emily A Ina, Shirley Ziton, Kirk Dourvetakis, Joseph P Corallo

**Affiliations:** 1 Osteopathic Medical School, Nova Southeastern University Dr. Kiran C. Patel College of Osteopathic Medicine, Fort Lauderdale, USA; 2 General Surgery, Broward Health Medical Center, Fort Lauderdale, USA; 3 General Surgery, Nova Southeastern University Dr. Kiran C. Patel College of Osteopathic Medicine, Fort Lauderdale, USA; 4 General Surgery, Broward Health and South Florida Surgical Specialists, Fort Lauderdale, USA

**Keywords:** antibiotic-resistant pathogens, colonic lavage, loop ileostomy, recurrent c. difficile, total colectomy

## Abstract

Fulminant *Clostridium difficile *colitis is a severe and potentially life-threatening form of *Clostridium difficile-*associated bacterial disease leading to inflammation and damage to the colon. Complications such as toxic megacolon, sepsis, and multi-organ failure commonly occur in individuals with compromised immune systems and recent antibiotic use. Management of *Clostridium difficile *colitis involves optimization of fluid and electrolyte balance, and elimination of bacteria commonly by administering vancomycin or fidaxomicin. In cases where pharmacological management has been ineffective, fecal microbiota transplantation and surgical intervention demonstrated success. Historically, surgical intervention has involved a total abdominal colectomy with end ileostomy; however, other surgical options have shown increasing benefits with preservation of the colon.

This case report aims to provide an example of an alternative management strategy for fulminant *Clostridium difficile *infections, via the use of a loop ileostomy and colonic lavage. The combination of loop ileostomy and colonic lavage promotes bowel rest, removes toxins, and promotes healing while decreasing inflammation. As with all management modalities, it is essential to recognize the associated complications. The potential benefits should be carefully weighed against the risks on a case-by-case basis with the help of a multidisciplinary team as illustrated through this case report. Overall, early recognition and treatment of fulminant *Clostridium difficile *colitis using loop ileostomy and colonic lavage prevents further disease progression and improves patient outcomes.

## Introduction

*Clostridium difficile (C. difficile)* is an anaerobic, gram-positive, spore-forming bacillus that impacts the gastrointestinal tract leading to profuse diarrhea and colonic inflammation. It is the most prevalent cause of antibiotic-associated nosocomial diarrhea with almost 3,000,000 *C. difficile* infections (CDIs) occurring every year in the United States [1]. Although recent antibiotic use is one of the most common risk factors for obtaining this infection, secondary factors include chemotherapy, inflammatory bowel disease, residence at a long-term care facility, and recent gastrointestinal procedures. The primary treatment for CDI is discontinuing the antibiotics that initially disrupted the normal colonic flora. In mild cases, metronidazole can be administered orally or intravenously. Oral vancomycin treatment is recommended if symptoms fail to improve or worsen after five to seven days of metronidazole [1,2]. Additionally, fidaxomicin is as effective as vancomycin, leading to significantly lower* C. difficile *recurrence rates [3].

Recurrent CDI occurs in 20-30% of patients and the risk of recurrence increases with age. However, it is difficult to distinguish a relapse with the same strain from reinfection by a new strain of *C. difficile* [1-4]. Treatment of recurrent infections may include tapered doses of vancomycin over 6 weeks, non-antibiotic treatments such as probiotics, intravenous immunoglobulins, and fecal microbiota transplantation [1]. Fecal microbiota transplantation has been found to be superior to fidaxomicin or vancomycin with a resolution rate of 70-90% in most patients [4]. 

When the symptoms continue to progress, leading to signs of end-organ dysfunction such as change in mental status, elevated lactate, or vasopressor requirement, surgical management is indicated [1,2]. The most commonly used procedure to manage fulminant *C. difficile* is a total abdominal colectomy with end ileostomy [5], however alternative surgical approaches, such as a diverting ileostomy, have gained increasing support [6].

This case report describes a 71-year-old female patient with an extensive medical history who presented with a fulminant CDI. Despite pharmacological management, she had a very high *C. difficile *recurrence rate due to which alternative management was required. Given the progression of her symptoms, she was deemed an appropriate candidate for surgical intervention. Rather than undergoing a total abdominal colectomy with end ileostomy, the patient was successfully managed with diverting loop ileostomy and colonic lavage. The aim of this case report is to illustrate the significance of having alternative management strategies for complex medical conditions and emphasize the crucial role that such approaches can play in mitigating morbidity and mortality in fulminant *C. difficile*.

## Case presentation

The patient was a 71-year-old female with an extensive medical history significant for recurrent *C. difficile*, anemia of chronic disease, metastatic uterine carcinoma status post total abdominal hysterectomy with chemoradiation, and obstructive uropathy requiring bilateral ureteral stent placement and dialysis. Initially, the patient was admitted to the hospital due to thrombosis of a tunneled dialysis catheter.

**Figure 1:**
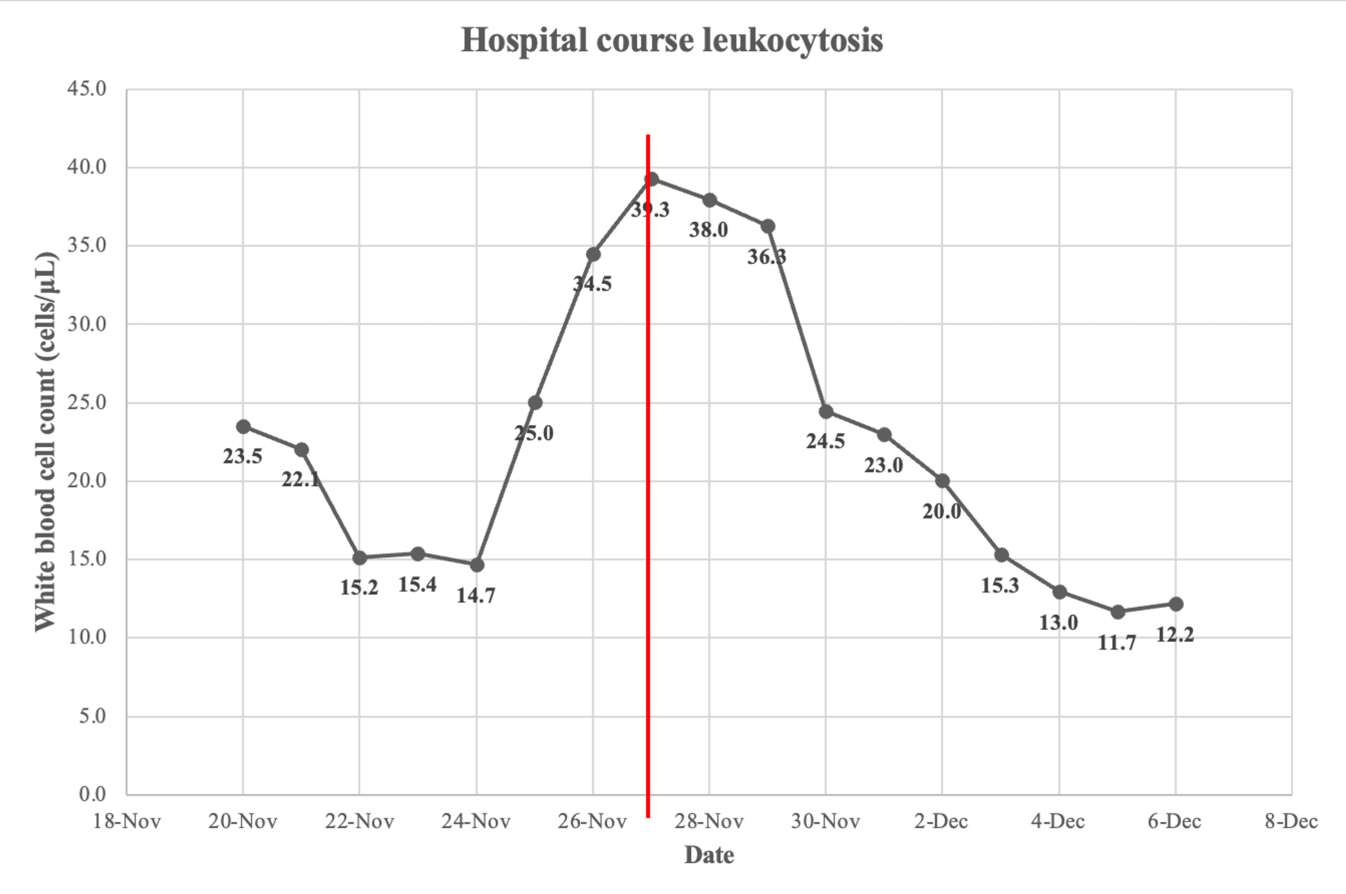
Hospital course of leukocytosis before and after intervention Red line: Date of surgical intervention.

The patient had a four-day history of diffuse abdominal pain which was worse in the left lower quadrant and the hypogastric region. She complained of associated nausea, vomiting, and an inability to tolerate food or water. She had four to five loose bowel movements on the day she was admitted. According to her medical records, the last episode of *C. difficile* was one month prior along with two previous episodes. After receiving intravenous fluids, transfused packed red blood cells, metronidazole 500 mg IV every six hours, and fidaxomicin 200 mg by mouth (PO) twice daily for several days, the patient’s diarrhea persisted, and her white blood cell count (WBC) continued to rise (Figure 1 and Table 1) leading to surgical consultation. The hospital course was additionally complicated by heparin-induced thrombocytopenia. Therefore, the patient was transferred to the intensive care unit (ICU) and argatroban was initiated.

**Table 1:** Hospital course of leukocytosis November 27: Date of surgical intervention

Date	White blood cell count (Reference range 4,500 to 11,000 cells/μL)
20-Nov	23.5
21-Nov	22.1
22-Nov	15.2
23-Nov	15.4
24-Nov	14.7
25-Nov	25.0
26-Nov	34.5
27-Nov	39.3
28-Nov	38.0
29-Nov	36.3
30-Nov	24.5
1-Dec	23.0
2-Dec	20.0
3-Dec	15.3
4-Dec	13.0
5-Dec	11.7
6-Dec	12.2

On initial evaluation, the patient was afebrile with a blood pressure of 119/60 mmHg, respiratory rate of 17 breaths/minute, and heart rate of 109 beats/minute. Physical examination revealed an ill-appearing female in moderate distress with mild abdominal distention and tenderness to palpation with voluntary guarding of left lower quadrant and suprapubic region. As summarized in Table 2, initial laboratory findings were significant for an elevated WBC at 23.5 x 10^3^ cells/µL (reference range 4,500 to 11,000 cells/μL), decreased hemoglobin at 8.9 g/dL (reference range 12-16 g/dL) with decreased hematocrit at 29.5% (reference range 36-48%). The complete metabolic panel also revealed decreased potassium levels of 2.6 mEq/L (reference range 3.5-5.5 mEq/L), most likely secondary to the patient’s persistent diarrhea.

**Table 2:** Initial laboratory findings

Laboratory parameter	Value	Reference range
White Blood Cell (cells/µL)	23.5x10^3^	4,500 to 11,000
Hemoglobin (g/dL)	8.9	12-16
Hematocrit (%)	29.5	36-48%
Potassium (mEq/L)	2.6	3.5-5.5

**Figure 2:**
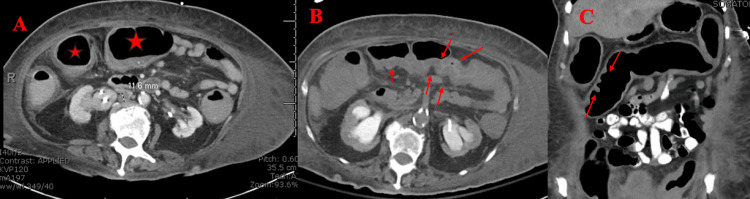
Abdominal CT scan A: Axial view with red stars indicating colonic dilation B: Axial view with red arrows indicating haustra effacement C: Sagittal view with red arrows indicating thumbprinting

Imaging studies included a computed tomography (CT) of the abdomen and pelvis with oral contrast which illustrated diffuse colonic dilation, diffuse wall thickening with thumbprinting, and haustral effacement indicative of possible toxic megacolon (Figures 2A-2C). Additionally, the CT revealed several hepatic masses, retroperitoneal lymphadenopathy, and bilateral iliac chain lymphadenopathy which was concerning for metastatic disease. 

The CT abdomen was repeated two days later and illustrated worsening colitis with concerns of toxic megacolon despite medical management with oral and rectal vancomycin, IV metronidazole, and fidaxomicin. Due to the lack of response from pharmacological therapy, it was determined that surgical intervention would offer the patient the best potential outcome and symptomatic relief. 

Given her previous hysterectomy, carcinomatosis, and metastatic disease, the common surgical approach of a subtotal colectomy was not recommended. After further consideration of the patient’s history and review of the imaging, she underwent an exploratory laparotomy with colon decompression and a loop ileostomy with transverse loop colostomy and colonic irrigation. 

Intraoperatively, carcinomatosis, diffusely dilated colon, ascites, and a large pelvic mass involving the terminal ileum and cecum were encountered. Given the large nature of the mass, a loop ileostomy proximal to the tumor was created with a postoperative plan to proceed with colonic antibiotic irrigation. Two red rubbers was placed, one each in the afferent and efferent limb. For 10 days postoperatively, irrigation of the afferent limb with vancomycin 500 mg and 100mL of NaCl 0.9% was performed every six hours via the red rubber. The efferent limb was irrigated with fidaxomicin 200 mg twice a day for five days through the other red rubber. Additionally, the patient received metronidazole 500mg every eight hours. Postoperatively, the patient remained afebrile, her leukocytosis improved and her diarrhea and abdominal pain resolved. Once the patient became hemodynamically stable and her laboratory values were closer to normal, she was transferred out of the ICU under the care of the internal medicine team. 

## Discussion

*C. difficile* is a major cause of healthcare-associated infection, particularly after disruption of the normal colonic flora through antibiotics, chemotherapy, and gastrointestinal procedures, among other causes [1]. Although typically resolved with oral and/or intravenous antibiotics, 3-10% of cases progress to severe, complicated, or fulminant colitis with a risk of bowel perforation, toxic megacolon, and death [1,6,7]. Due to the high mortality of fulminant CDI, early surgical intervention is recommended [6,7]. However, since only 30% of patients with severe CDI will require emergent surgical intervention, there is a risk of over-treatment with an early surgical approach [7,8]. Moreover, the timing of the optimal therapeutic window is complicated by the high mortality and morbidity associated with total colectomy with end ileostomy, which has been the gold standard for surgical treatment of fulminant CDI refractory to medical treatment. 

Alternative approaches have been evaluated to reduce mortality and morbidity associated with fulminant *C. difficile*. Diverting loop ileostomy (DLI) with irrigation and lavage of the colon has been proposed as an alternative for fulminant CDI [6,8]. A case series done by Neal et al. established the Pittsburgh protocol. This novel technique showed that total colectomy can be avoided in complex patients at a high risk of developing severe *C. difficile* colitis through early intervention with loop ileostomy and colonic lavage [9]. Though Neal et al. showed a significant reduction in morbidity and mortality compared to the standard surgical approach, the results have faced scrutiny for shortcomings in the study design. Moreover, follow-up studies have failed to replicate the significant reduction in mortality measured in this study [6,10]. A systematic review and meta-analysis noted that outcomes following emergency surgery for *C. difficile* colitis supported total colectomy with end ileostomy as the primary surgical treatment. However, it conceded that less extensive surgery, such as loop ileostomy with colonic lavage, may be a viable alternative intervention for certain populations in the early stages [11,12]. A more recent study using a propensity-matched analysis compared total abdominal colectomy and partial colectomy, and found that the latter did not increase mortality [5]. The literature suggests that further evidence is needed to refine optimum treatment alternatives in the surgical approach to fulminant CDI.

The presented case highlights the challenges and complexities associated with fulminant CDI, particularly in a patient with a significant medical history and risk factors. The patient, a 71-year-old female, had a background of recurrent *C. difficile*, metastatic uterine cancer, obstructive uropathy, and bilateral ureteral stent placement. The initial presentation with thromboses of the tunneled dialysis catheter, diffuse abdominal pain, and persistent diarrhea set the stage for a complex medical scenario. Instead of the more common total abdominal colectomy with end ileostomy, this case illustrates the benefits of diverting loop ileostomy with colonic lavage, using a variation of the Pittsburgh protocol. This approach was chosen to divert stool, minimize the risk of future obstructions, and provide targeted treatment through afferent and efferent limb irrigation. 

The unique aspect of this case lies in the choice of surgical intervention, a DLI, coupled with a protocol for colonic lavage for the treatment of fulminant *C. difficile* colitis. The Pittsburgh protocol utilized loop ileostomy with intraoperative lavage using polyethylene glycol (PEG) and electrolyte infusion, as well as postoperative antegrade vancomycin flushes via the ileostomy and IV metronidazole [6]. A pilot study proposed by McCreery et al. suggests a protocol of intestinal lavage using bedside PEG via a nasogastric tube in addition to usual antibiotic treatment instead of a surgical approach in severe, complicated CDI [7]. Another case report followed a similar treatment as used in the Pittsburgh protocol, but used a MIC gastrostomy tube rather than a Malecot drain, a variation that the authors claimed provided a more efficient administration [9]. The treatment in the present case following loop ileostomy included irrigation of both the afferent and efferent limbs with vancomycin and fidaxomicin respectively. Additionally, the patient was treated with metronidazole. Following surgery, her clinical course showed improvement. She was afebrile postoperatively, the WBC count began to decrease, and persistent diarrhea with abdominal pain resolved. The use of vancomycin and fidaxomicin for irrigation in the postoperative period highlights a tailored approach to address the CDI at the site of concern.

This case underscores the significance of considering alternative approaches in managing fulminant CDI. While surgical intervention is indicated in fulminant cases, and total abdominal colectomy is a standard procedure, the high mortality associated with total colectomy requires improvement in the treatment protocol. The choice of a DLI in this case illustrates the importance of individualized treatment strategies. An approach tailored to the patient’s specific condition can play a crucial role in mitigating morbidity and mortality associated with complex cases of fulminant *C. difficile* colitis.

## Conclusions

In conclusion, this case report provides valuable insights into the multifaceted challenges of managing fulminant CDI in a patient with a complex medical history. The use of a DLI coupled with colonic lavage demonstrates the importance of adapting surgical strategies to individual patient circumstances, highlighting the need for alternative approaches in managing challenging clinical scenarios. Continued collaboration, ongoing research, and adherence to evidence-based practices are crucial for further improving outcomes in patients with severe fulminant CDIs. Prospective studies are needed to evaluate outcomes after loop ileostomy and lavage for treating this potentially life-threatening disease.
